# Pharmacies as providers of expanded health services for people who inject drugs: a review of laws, policies, and barriers in six countries

**DOI:** 10.1186/1472-6963-14-261

**Published:** 2014-06-17

**Authors:** Theodore M Hammett, Son Phan, Julia Gaggin, Patricia Case, Nicholas Zaller, Alexandra Lutnick, Alex H Kral, Ekaterina V Fedorova, Robert Heimer, Will Small, Robin Pollini, Leo Beletsky, Carl Latkin, Don C Des Jarlais

**Affiliations:** 1 Abt Associates Inc., Cambridge MA 02138, USA; 2 Abt Associates Inc., Bethesda MD 20814, USA; 3 The Fenway Institute, Fenway Health, Boston MA 02215, USA; 4 The Miriam Hospital, Center for AIDS Research, Providence RI 02906, USA; 5 RTI International, San Francisco CA 94103, USA; 6 The Biomedical Center, St. Petersburg 194044, Russia; 7 Yale University School of Public Health, New Haven CT 06520, USA; 8 British Columbia Centre for Excellence in HIV/AIDS, Vancouver BC V6Z 1Y6, Canada; 9 Faculty of Health Sciences, Simon Fraser University, Burnaby, BC, Canada; 10 Pacific Institute for Research and Evaluation, Calverton MD 20705, USA; 11 Northeastern University School of Law & Bouvé College of Health Sciences, Boston MA 02144, USA; 12 Johns Hopkins Bloomberg School of Public Health, Baltimore MD 21205, USA; 13 Beth Israel Medical Center, New York NY 10038, USA

## Abstract

**Background:**

People who inject drugs (PWID) are underserved by health providers but pharmacies may be their most accessible care settings.

**Methods:**

Studies in the U.S., Russia, Vietnam, China, Canada and Mexico employed a three-level (macro-, meso-, and micro-) model to assess feasibility of expanded pharmacy services for PWID. Studies employed qualitative and quantitative interviews, review of legal and policy documents, and information on the knowledge, attitudes, and practices of key stakeholders.

**Results:**

Studies produced a mixed assessment of feasibility. Provision of information and referrals by pharmacies is permissible in all study sites and sale and safe disposal of needles/syringes by pharmacies is legal in almost all sites, although needle/syringe sales face challenges related to attitudes and practices of pharmacists, police, and other actors. Pharmacy provision of HIV testing, hepatitis vaccination, opioid substitution treatment, provision of naloxone for drug overdose, and abscess treatment, face more serious legal and policy barriers.

**Discussion:**

Challenges to expanded services for drug users in pharmacies exist at all three levels, especially the macro-level characterized by legal barriers and persistent stigmatization of PWID. Where deficiencies in laws, policies, and community attitudes block implementation, stakeholders should advocate for needed legal and policy changes and work to address community stigma and resistance. Laws and policies are only as good as their implementation, so attention is also needed to meso- and micro- levels. Policies, attitudes, and practices of police departments and pharmacy chains as well as knowledge, attitudes, and practices of individual PWID, individual pharmacies, and police officers should support rather than undermine positive laws and expanded services. Despite the challenges, pharmacies remain potentially important venues for delivering health services to PWID.

## Background

Almost 150 countries on all continents had reported injection drug use by 2007 and 120 of these had reported related HIV infections [1]. According to estimates for 2010–2011, HIV prevalence among the 14–16 million people who inject drugs (PWID) worldwide is 12% with wide variation across regions [2,3]. Injection drug use is driving HIV epidemics in many countries in Asia, the Middle East, and Eastern Europe and there are continuing epidemics of HIV and other parenterally transmitted viruses such as hepatitis C among PWID in many locations worldwide [4-6]. In many countries, controlling HIV/AIDS and hepatitis epidemics depends on reducing transmission among PWID and other people who use drugs.

For various reasons including widespread stigma and discrimination, people who use drugs are generally underserved by and/or reluctant to visit traditional health providers – physicians, health clinics, and hospitals [7]. In developed countries, many drug users rely on paramedic and/or emergency room care when they are seriously ill, suffer overdose, or experience other health problems. In both developed and developing countries, pharmacies are a type of health facility that is commonly accessed by drug users but their use is often limited to the purchase of needles/syringes and other items related to drug injection [8-10]. Because drug users frequently visit pharmacies there is the potential for pharmacies to provide expanded health services for them [11].

To assess the potential for pharmacies to become venues for expanded health services for PWID, including services related to HIV/AIDS, hepatitis, and substance use prevention and treatment, the National Institute on Drug Abuse, U.S. National Institutes of Health funded linked grants from a consortium of investigators from six sites in four countries – the United States (Boston, Providence, and San Francisco), Russia (St. Petersburg), Vietnam (Ha Giang City), and China (Xichang, Sichuan Province) – to conduct mixed-methods feasibility studies. These sites were selected to provide a range of geographic settings and perceived attitudes towards harm reduction and other services for drug injectors.

The feasibility studies were carried out between 2009 and 2012 with investigators conducting similar studies in Vancouver, Canada and Tijuana, Mexico joining the research group in 2011, bringing the number of countries represented to six and the number of sites to eight. Table 1 presents basic statistics on the epidemics of injection drug use and HIV in these eight sites.

**Table 1:** Injection drug use, HIV, and research methods in study sites

**Site**	**Est. n PWID**	**Predominant injected drug(s)**	**Est. HIV prevalence among PWID**	**Est. % of HIV infections attributable to drug use**	**Methods**
*Original sites funded under linked NIDA grants*					
**Boston**	10,064^a^	heroin	4.5%^b^-8.4%^c^	9%^d^	Formative research, qualitative interviews, quantitative surveys, review of legal and policy documents
**Providence**	2,137^a^	opiates	5.3%^b^	6%^e^	
**San Francisco**	17,000	heroin, methamphetamine	12%	22%^f^	
**Ha Giang, Vietnam**	1,000	heroin	18%	>50%	
**St. Petersburg, Russia**	83,000^g^	heroin	44%-59%^h^	76%	
**Xichang, China**	2,250	heroin	18%	60%	
*Additional sites*					
**Tijuana, Mexico**	6,400-10,000	heroin, heroin/methamphetamine	4%^i^	12%	Focus groups, quantitative survey, review of legal and policy documents
**Vancouver, Canada**	10,000-15,000	Cocaine, heroin, methamphetamine	17% (2006)	18% (since 2008)	Ongoing ethnographic and qualitative research, review of legal and policy documents

^a^Based on methods in Brady JE, Friedman SR, Cooper HL, Flom PL, Tempalski B, Gostnell K. **Estimating the prevalence of injection drug users in the U.S. and in large U.S. metropolitan areas from 1992 to 2002**. *J Urban Health* 2008, **85**: 323–351.

^b^Tempalski B, Lieb S, Cleland CM, Cooper H, Brady JE, Friedman SR. **HIV prevalence rates among injection drug users in 96 large US metropolitan areas, 1992–2002**. *J Urban Health* 2009, **86**: 132–154.

^c^National Health Behavior Survey, 2009.

^d^Cases reported in 2010, from Massachusetts Department of Public Health, 2012 Epidemiological Profile.

^e^Cases reported 2008–2010, from Rhode Island Department of Public Health, 2010 Epidemiological Profile.

^f^Newly reported cases in 2010, includes PWID and MSM/PWID.

^g^Heimer R, White E. **Estimation of the number of injection drug users in St. Petersburg, Russia**. *Drug Alcohol Dependence* 2010, **109**: 79–83.

^h^Niccolai LM, Shcherbakova IS, Toussova OV, Kozlov AP, Heimer R. **The potential for bridging of HIV transmission in Russian Federation: sex risk behaviors and HIV prevalence among drug users (DUs) and their non-DU sex partners**. *J Urban Health* 2009, **86**(Suppl 1): 131–143; Eritsyan K, Heimer R, Barbour R, Odinokova V, White E, Rusakova MM, Smolskaya TT, Levina OS. **Individual-, network-, and city-level factors associated with HIV prevalence among people who inject drugs in eight Russian cities**. *BMJ Open*, In press.

^I^Strathdee SA, Lozada R, Ojeda VD, Pollini RA, Brouwer KC, Vera A, Cornelius W, Nguyen L., Magis-Rodriguez C, Patterson TL, Proyecto El Cuete. **Differential effects of migration and deportation on HIV infection among male and female injection drug users in Tijuana, Mexico**. *PLoS One* 2008, **3**(7):e2690.doi:0.1371/journal.pone.0002690.

The projects aimed to assess current pharmacy services and the potential for their expansion and to identify barriers to and facilitators of such expanded services. These factors included pharmacists’ attitudes and possible interference from law enforcement and policy makers. In this paper, we assess further the feasibility of pharmacies to implement expanded services for PWID based on the legal and policy frameworks of the six countries and other inhibiting and facilitating factors.

## Methods

The multi-site feasibility studies of expanded pharmacy services for drug users employed mixed methods: qualitative and quantitative interviews with key stakeholders in health, police, and other sectors, including government officials, health care providers, pharmacists, and PWID, as well as review of relevant legal and policy documents and literature in the field.

The original NIDA grants supported formative study, qualitative interviews, and quantitative surveys in the six original sites. The formative study described the IDU and HIV epidemic situations. Qualitative interviews were then conducted with PWID and key informants from legal, public health, and pharmacy systems and quantitative face-to-face surveys were conducted among PWID and pharmacists. All data collection investigated current HIV and substance abuse services for PWID and the needs for additional services, explored perceptions of pharmacists regarding new services for PWID, and identified barriers to pharmacy participation in health-related interventions for PWID, including possible interference from law enforcement and policy makers. To explore further the enabling environment for potentially expanded pharmacy-based interventions, each site investigator reviewed relevant legal and policy documents and corporate policies of pharmacy chains bearing on the implementation of the expanded services identified in the feasibility studies. The site investigators also provided information from their own knowledge on street-level practices that conform to or contradict the laws and policies of governments and pharmacy chains.

Investigators in the additional sites employed slightly different methods. In Tijuana, information was obtained through focus group discussions and a quantitative survey, as well as review of legal and policy documents. In Vancouver, review of relevant legal and policy documents and ongoing ethnographic and qualitative research by the site investigator were used.

To inform further the analysis of these data, we adapted a conceptual framework based on elements proposed by Rhodes [12,13] and Burris [14]. This framework consists of three levels, defined as follows:

• Macro level: Laws and policies governing pharmacy operations and procedures; overall legal framework on drugs and drug paraphernalia; attitudes of the general community;

• Meso level: Attitudes, policies and practices of pharmacy chains and police agencies; and

• Micro level: Behavior and attitudes of individual pharmacies/pharmacists, police officers, and people who inject drugs.

## Results

The feasibility studies found that the following types of services were most desired by PWID and most likely to be offered by pharmacies in the future: expanded needle/syringe sales and distribution; safe disposal of used needles/syringes; HIV testing; hepatitis B and other vaccinations; methadone maintenance and/or other opioid substitution treatment; provision of naloxone for overdose prevention and rescue; skin abscess prevention and treatment; brief counseling and informational materials; and referrals to HIV/AIDS, substance abuse treatment, and other services.

Table 2 summarizes the government laws and policies in the six countries that are most relevant to each of the proposed expanded services. Table 3 summarizes pharmacy chains’ policies in the sites where such chains exist and information on their policies was available: Boston, Providence, San Francisco, Vancouver, and St. Petersburg.

**Table 2:** Government laws and policies

**Sites: Interventions:**	**US (Boston)**	**US (Providence)**	**US (San Francisco)**	**Canada (Vancouver)**	**Mexico (Tijuana)**	**Vietnam (Ha Giang)**	**China (Xichang, Sichuan)**	**Russia (St. Petersburg)**
**Needle & syringe sales/distribution**	MGL ch 94C, sec 27, 27A, 32 L: authorizes pharmacy sales without prescription at discretion to people 18 or older with no limits on quantity	Pharmacies may sell at discretion; no limits on quantity.	Non-prescription sales legal (up to 30) but requires local approval and pharmacist enrollment in disease prevention demonstration project (DPDP).	Pharmacy sales legal.	Sale at pharmacies without a prescription is legal.	Retail non-prescription sale is legal; redemption of vouchers for free needles & syringes also legal.	Non-prescription sale is legal.	Non-prescription sale is legal; but pharmacies not required to stock.
**Needle & syringe disposal**	MGL ch 111, sec 127A: prohibits improper disposal. There are no legal barriers to pharmacies collecting waste for proper disposal.	No legal barrier to pharmacies maintaining sharps containers.	Sharps must be disposed in approved container and transported to collection center; no legal barriers to sharps containers in pharmacies; information on safe disposal required to be provided with needle/syringe sales under DPDP; participating pharmacies must provide safedisposal options.	No legal barriers to pharmacies collecting used needles/syringes.	Any facilities (including pharmacies) that generate at least 25 kg of biologic/infectious waste per month are required to dispose of syringes and other sharps in a puncture-proof container. Unclear what rules apply to pharmacies generating less than 25 kg per month.	Not provided for in Law on Pharmacy (2005); but Ordinance on Private Medical and Pharmaceutical Practice (Feb 25, 2003) authorizes those certified as private medical practitioners (including those with pharmacy degrees) to provide primary health care and HIV prevention services (Art. 18).	Needle exchange and disposal center can be set up in local CDC, medical institutes or other locations. No legal barriers for pharmacies to provide sharps containers.	Federal Law #128 (August 8, 2001) requires a license for collection, storage, or disposal of hazardous waste (including used needles & syringes).
**HIV testing**	There is currently no standing order for pharmacists to conduct HIV testing.	Pharmacies need CLIA waiver to offer finger prick testing.	Persons with required training & certification may perform rapid HIV tests; regulations would need to be modified to allow pharmacies to draw blood, but not if testing was done by an outside organization.	Provincial requirements for pre-/post-test counseling may constrain pharmacy-based testing.	Cheek-swab tests possible but no blood may be drawn or handled at pharmacies.	Not provided for in Law on Pharmacy (2005); may be permitted by Ordinance on Private Medical and Pharmaceutical Practice (Art. 18), if pharmacies can be considered medical establishments.	Only local HIV/AIDS prevention agencies may do testing; pharmacies not included.	Decree #1081 (December 22, 2011) on licensing of pharmacies does not allow pharmacies to conduct HIV testing; additional license for medical practice is required (FL #128 of August 08, 2011; Decree #30 of January 22, 2007).
**HBV/other vaccination**	Critical adult vaccinations permitted by pharmacists following appropriate training.	No legal barriers; other vaccinations already available in pharmacies.	HBV vaccination required for children; Persons with required training and certification may provide vaccination.	No legal barriers to pharmacies providing HBV or other vaccinations. However, it would require staff who have appropriate training, and adequate staffing levels.	The law does not permit vaccinations at pharmacies unless the staff has the proper certification, such as a nursing degree.	Not provided for in Law on Pharmacy (2005); may be permitted by Ordinance on Private Medical and Pharmaceutical Practice (Art. 18) if pharmacies can be considered medical establishments.	Vaccination is not allowed in pharmacies. Vaccination requires licensed medical doctor, medical assistant, or nurse and infrastructure to store vaccine.	Decree #1081 (December 22, 2011) on licensing of pharmacies does not allow pharmacies to provide vaccinations; additional license for medical practice is required (FL #128 of August 08, 2011; Decree #30 of January 22, 2007).
**MMT/OST**	Federally regulated; methadone for drug treatment cannot be dispensed in pharmacies.	Federally regulated; methadone for drug treatment cannot be dispensed in pharmacies.	Office-based physicians can prescribe methadone and prescriptions are treated like any other but most pharmacies are not equipped to do daily dosing.	Pharmacies can dispense methadone, but regulations restrict prescription.	Methadone is a “Control 1” drug that requires a prescription limited to one-time administration, which effectively bars daily dosing, and “Control 1” drugs can only be handled by major medical institutions.	Prohibited by Law on Pharmacy (2005) Article 26.2 because methadone is listed as a habit-forming drug, which pharmacies are prohibited from dispensing.	Methadone is strictly controlled and can only be dispensed in government approved drug treatment centers. It cannot be dispensed in pharmacies.	Methadone is included in List I of forbidden psychoactive agents (FL on narcotics and psychoactive agents of December 10, 1997).
**Naloxone/overdose prevention/rescue**	Pharmacies may dispense through standing orders; training is needed on nasal delivery.	Pharmacies may dispense through collaborative practice agreements	Limited liability for licensed health providers prescribing and/or distributing naloxone.	Regulatory barriers make naloxone unavailable to individuals through prescription.	Pharmacies stock naloxone as a “Control 3” drug that requires a prescription that may be filled 3 times and expires in 6 months.	Not provided for in Law on Pharmacy (2005); may be permitted by Ordinance on Private Medical and Pharmaceutical Practice (Art. 18), if pharmacies can be considered medical establishments.	Naloxone can only be dispensed in medical facilities. Print materials on overdose prevention can be provided in pharmacies.	Decree #2199-r (December 7, 2011): naloxone is on the list of vitally necessary and most essential medications; but only medical facilities are allowed to dispense and administer and to provide CPR if needed.
**Abscess treatment**	Not currently available but no legal barriers to providing information. Pharmacies cannot provide medical or clinical care such as cutting and draining.	Not currently available but no legal barriers to providing information. Pharmacies cannot provide medical or clinical care such as cutting and draining.	No legal restrictions.	No legal barriers.	No legal barriers, but only minor wounds may be treated at a pharmacy. Perforation or cutting of any sort in order to treat an original wound not permitted.	Not provided for in Law on Pharmacy (2005); may be permitted by Ordinance on Private Medical and Pharmaceutical Practice (Art. 18), if pharmacies can be considered medical establishments.	Pharmacies can provide print materials only.	Pharmacies can provide information and medications.
**Brief counseling/materials**	No legal barriers except to substance abuse treatment counseling, which is covered by state regulations	No legal barriers.	No legal barriers; DPDP participating pharmacies required to provide health information with needle & syringe sales	No legal barriers.	No legal barriers.	Should be permissible.	Should be permissible.	Pharmacies can provide such information.
**Referrals**	No legal restrictions.	No legal restrictions.	No legal restrictions.	No legal restrictions.	No legal restrictions.	No legal restrictions.	No legal restrictions.	No legal restrictions.

**Table 3:** Corporate pharmacy policies (chain pharmacies)

***Sites/interventions***	**US (Boston)**	**US (Providence)**	**US (SF)**	**Canada (Vancouver)**	**Russia (St P)**
**Needle/syringe sales/distribution**	Corporate policy either specifically allows for or is silent on non-prescription sales of syringes. Most pharmacies sell needles/syringes but the law provides that sales of syringes is at the discretion of the pharmacist.	Sales may be at the discretion of the on-site pharmacy manager	Chains can enroll in Disease Prevention Demonstration Programs so individual stores can sell needles/syringes, but implementation within chains has been inconsistent.^a^	Most chains sell needles/syringes but individual stores can set policies; many stores will not sell	Chains have no objection to selling needles/syringes but would probably oppose free distribution
**Needle/syringe disposal**	Pharmacies generally will not accept used syringes for disposal.	Pharmacies generally will not accept used syringes for disposal.	One chain allows; another prohibits	Most chain pharmacies have sharps containers and accept full containers; individual store policies may differ	Requires additional license for collecting and storage of epidemiologically hazardous waste
**HIV testing**	HIV testing is not available in pharmacies.	HIV testing is not available in pharmacies.	Some pharmacies in one chain offered free HIV testing as part of National HIV testing month	Not currently available	Only at medically-licensed facility
**HBV/other vaccination**	A recent change in pharmacy policy (MDPH, Drug Control Program and Immunization Program, Joint Policy 2012–2) permits administration of the hepatitis B vaccine in pharmacies to adults by a qualified pharmacist. Two major corporate chains have opened walk in clinics for adult vaccination including hepatitis B vaccines.	Pharmacies may offer any vaccine including hepatitis B, pneumonia, shingles, pertussis, tetanus, meningitis and human papillomavirus.	Most chains offer vaccinations	Could be offered if staff are properly trained	Only at medically-licensed facility
**MMT/OST**	Federal Law prohibits dispensing methadone at retail pharmacies.	Federal Law prohibits dispensing methadone at retail pharmacies.	Prescriptions could be filled but DOT requires corporate approval	Regularly dispensed by prescription	Methadone is a prohibited psychoactive agent
**Naloxone/overdose prevention/rescue**	Prescriptions for naloxone may be filled.	Also see collaborative practice agreement comments above (Table 2).	Prescriptions could be filled	Individuals cannot obtain by prescription; pharmacies fill orders for clinics and other authorized organizations	Not generally stocked by pharmacies
**Abscess treatment**	Corporate policies support provision of information	Corporate policies support provision of information	Information about wound care can be provided	Pharmacies refer customers to health clinic	Pharmacies could offer information; requires display space
**Brief counseling/materials**	Corporate policies support the provision of information.	Corporate policies support the provision of information.	Information about medications and health concerns can be provided	Some pharmacies may have informational materials, but most do not	Pharmacies could offer information; requires display space
**Referrals**	Corporate policies support the provision of information.	Corporate policies support the provision of information.	Referrals can be made	Pharmacies make referrals	Pharmacies could offer

^a^Lutnick A, Cooper E, Dodson C, Bluthenthal R, Kral AH. **Pharmacy syringe purchase test of nonprescription syringe sales in San Francisco and Los Angeles in 2010**. *J Urban Health* 2012.doi:10.1007/s11524-012-9713-7.

Needle/syringe sales without prescription are legal in all study sites, but several have experienced practical barriers to implementation including attitudes and behaviors of individual pharmacies, even if the law or their chains authorize sales. In Vietnam, a female injector said that

pharmacy sellers should be hospitable, because…IDUs are usually ashamed… If you’re not a hospitable pharmacy seller, then here come a bad IDU [and] he/she could shout at you …--49 year-old ethnic female PWID, injecting for 11 years

In Tijuana, where pharmacy sales of needles/syringes are legal, focus group discussions with PWID revealed that many pharmacies refused to sell needles/syringes due to negative attitudes towards drug users, fear that PWID behavior would drive away other customers, and concern about police response [15]. In a “buy study”, only 28% of attempted purchases by “mystery shoppers” were successful [16]. Tijuana PWID noted the unpredictability and inconsistency of pharmacy sales and the commonly negative attitudes of pharmacy workers:

I mean it can’t be distinguished as one pharmacy or another, the one that does sell to you and the one that doesn’t. You go into the pharmacy and there are times when it does sell to you and others when it doesn’t. – Male focus group participant.

[W]e’re “tecatos”,….we’re junkies and drug addicts, many pharmacies don’t want to sell it to you…. I’ve noticed by how they look at us. –Female focus group participant.

In San Francisco, under a Disease Prevention Demonstration Project (DPDP), participating pharmacies may sell up to 30 needles/syringes at a time but a “buy study”, carried out as part of the feasibility study presented in this paper, revealed inconsistent compliance with program provisions including outright refusals to sell and varying minimum numbers of needles/syringes required for purchase [17].

In St. Petersburg, Russia, a syringe “buy study”, also conducted as part of the pharmacy feasibility study in one central and one peripheral district of the city, found that half of the pharmacies that refused to sell syringes to research staff nonetheless reported in a telephone survey that they stocked syringes [18].

There appear to be no legal barriers to needle/syringe disposal in pharmacies except in St. Petersburg where a special license for such disposal is required, and Ha Giang, Vietnam where disposal of used needles/syringes at pharmacies must comply with the Ministry of Health’s regulations on management of medical wastes (Vietnam Ministry of Health. Decision No. 43/2007/QD-BYT, November 30, 2007). However, individual pharmacies might refuse to offer disposal services. Cost may also play a role in such decisions, as revealed in St. Petersburg:

It [syringe disposal] costs a lot. Not every medical facility can afford waste disposal. --Department Head, drug treatment facility

In San Francisco, some pharmacies’ reluctance to offer required disposal services may explain their refusal to join the DPDP, under which needles/syringes are sold without prescription [17]. In Massachusetts, there is no barrier to pharmacies accepting syringes for disposal, but very few do, citing the high cost and safety concerns with handling medical waste. The Massachusetts Department of Public Health provides disposal “kiosks” but none of these are placed in pharmacies. Biohazardous waste containers and prepaid envelopes to mail used syringes to a biohazardous waste company may be purchased in some pharmacies.

In most sites, HIV testing in pharmacies is not legally barred but cannot be provided without additional staff training or certification. In Tijuana, only saliva-based testing may be done in pharmacies because blood drawing is prohibited. Similarly, St. Petersburg has an explicit prohibition on blood/serum-based HIV testing in pharmacies since this practice is restricted to licensed medical facilities. Oral testing could be conducted at pharmacies in St. Petersburg but individuals testing positive would require confirmatory testing by the Municipal AIDS Center to qualify for care and treatment. In Vancouver, strict requirements for provision of pre/post-test counseling limit the potential for offering HIV testing in pharmacies, although access to testing has recently been expanded through health clinics and community health centers.

In Boston, a recent change in pharmacy regulations permits walk-in clinics to offer adult immunization, including for hepatitis B. With the walk-in clinic infrastructure in place, adding HIV testing may be feasible, but is not currently permitted. Some pharmacy chains operating in San Francisco allow vaccinations but others do not. Several sites, including Boston, San Francisco, Tijuana and Vancouver, require special training or certification for staff to administer vaccinations, while in St. Petersburg pharmacies are prohibited from administering any vaccinations since this requires a medical license and separate space in the facility:

[Vaccination against hepatitis) is impossible [in pharmacies] due to technical reasons. There are numerous regulations that must be met according to the “Sanitary Rules and Norms.” For instance, there must be a separate room with specific technical devices –Deputy Director, drug treatment facility.

Provision of methadone or other OST in pharmacies faces serious legal and practical barriers. A pharmacy worker in Ha Giang, Vietnam noted that

Vietnam Ministry of Health should enact legal documents so we are allowed to sell… it will be troublesome if selling is not permitted. Some specific drugs only university-leveled pharmacists are permitted to sell”. --pharmacy worker in Minh Khai ward

A number of the sites prohibit provision of OST in pharmacies, while in several others special legal dispensation is needed for prescribing methadone or similar medications. In Russia, standard OST is prohibited by law in all facilities. In Vancouver, OST in pharmacies is legally permissible but a substantial proportion of methadone patients attend “private methadone clinics” [19]. In addition, Vancouver physicians must receive an exemption under the Controlled Drugs and Substances Act to prescribe methadone or other medications used in OST.

The provision by pharmacies of naloxone for overdose prevention and rescue also confronts diverse legal and practical challenges. The difficulty or impossibility of PWID getting to pharmacies to receive naloxone while suffering from overdose has prompted the creation of community-based overdose education and naloxone access efforts in some places, designed to increase the likelihood of timely emergency response [20]. Across all sites, naloxone remains a prescription drug, used primarily in clinical settings and by emergency medical staff and police, as recently proposed by New York’s Attorney General [21]. Naloxone is seldom stocked in pharmacies. This inhibits efforts to utilize pharmacies as access points for PWID and their caregivers [22].

In Boston, naloxone could be provided to PWID by pharmacists under the existing “standing order”. This order, issued in 2007 by the medical director of the overdose prevention program, provides that community members and agency workers may be trained to provide nasal naloxone to drug users for overdose prevention. In Providence, naloxone may be provided by pharmacies under collaborative practice agreements with prescribers as part of wider overdose prevention and naloxone access schemes [23]. A collaborative practice agreement is a signed agreement, entered into voluntarily, between a pharmacist with advanced training and experience relevant to the scope of collaborative practice and one or more physicians that defines the collaborative pharmacy practice in which the pharmacist and physician(s) propose to engage.

Many San Francisco pharmacists said that they would routinely fill prescriptions for naloxone. Under California law (SB 767 [2007]), licensed health providers who prescribe and/or distribute naloxone for emergency treatment of drug overdose bear only limited liability. In 2010, this limitation on liability was extended to lay providers of naloxone who have received appropriate training through a program registered by a local health department [http://legiscan.com/CA/text/AB2145/id/60864].

In St. Petersburg, naloxone may be dispensed and administered only in licensed health care facilities. However, naloxone is included in the “List of vitally necessary and the most essential medicinal agents for 2012” (*Decree of the Government of RF#2199-r dated of December 7, 2011*) which ensures “physical and economical availability” of certain medicinal products. Thus, pharmacies could be compelled to make naloxone available should the appropriate government agencies wish to promote its availability. However, according to one drug treatment professional:

Although it should be legalized, as far as I know, narcologist cannot prescribe naloxone to a drug addict. …There is no indication in the labeling for naloxone that would allow it to be used for overdose prevention outside the clinical setting. It says that [the] medication is to be used in medical facilities by a physician. –Department Head, drug treatment facility

According to laws or regulations in most sites, pharmacists cannot clean or dress abscesses because this would constitute a practice of medicine prohibited to them. In some countries, as well, providing such care could also raise medical malpractice issues. However, in most sites pharmacists could dispense antibiotics or ointments for treatment of abscesses, with or without a prescription depending on the country. In San Francisco, abscess treatment in pharmacies may not be authorized by relevant government agencies because such services are already available at clinics and hospitals.

There are few if any legal or policy barriers to pharmacies providing information, materials, brief counseling and referrals on any health issues, including HIV/AIDS, hepatitis, abscess treatment, overdose prevention and rescue, and substance abuse treatment. The challenges that exist arise largely from the practices and attitudes of individual pharmacies and their staffs. In San Francisco, the needle/syringe “buy study” found that many pharmacies participating in the DPDP were not providing written or oral information on substance abuse treatment, HIV/HCV testing, and safe disposal of used needles/syringes that is required to accompany each needle/syringe sale [17].

## Discussion

This review of laws, policies, and practices presents a mixed picture of the possibilities for implementing pharmacy-based service enhancements for drug users. Provision of informational materials and referrals in pharmacies is legally permissible in all study sites and sale and safe disposal of needles/syringes by pharmacies is legal in almost all sites. Pharmacy provision of other services identified as most promising in the study, such as HIV testing, hepatitis and other vaccinations, opioid substitution treatment, provision of naloxone for treatment of opioid overdose, and abscess treatment face more serious and pervasive legal and policy barriers.

We identified challenges to achieving a truly enabling environment for the implementation of expanded pharmacy services at all three levels of our conceptual model. The most commonly identified challenges occur at the macro-level where legal and policy provisions block provision of some services and medications in pharmacies and persistent stigma and its internalization by PWID reduce uptake of services that do exist. Lack of relationships between pharmacies and HIV prevention and addiction-related resources in the community and consequent lack of consensus as to who should provide specific HIV-related services also represent barriers to expansion of services in pharmacies. In places dominated by private chain pharmacies, skepticism that providing additional services for PWID would translate into increased pharmacy profits have also hindered offering such services.

At the meso-level, our study uncovered variations in policy and practice related to services for PWID across police agencies and pharmacy chains. For example, some pharmacy chains narrowly define pharmacists’ roles and responsibilities and limit interventions. Lack of sufficient time and appropriate space may also limit possibilities for expansion of services in pharmacies.

At the micro-level, differences in knowledge (such as lack of understanding of addiction and harm reduction), attitudes (such as stigmatizing PWID customers), and behaviors (such as refusal to sell needles/syringes to suspected PWID) of individuals pharmacists, pharmacy workers, shift managers in large pharmacies, police officers, and other actors pose barriers to the expansion of pharmacy-based services for PWID. In Russia, for example, all aspects of life are highly bureaucratized and officials and pharmacists feel that they must comply with all regulations or risk punishment and damage to their careers. They may also use the existence of prohibitory rules and regulations to rationalize the denial of services that they know or suspect would advance public health.

At the macro- level, where deficiencies and barriers in governmental laws and policies may block implementation, stakeholders should advocate with relevant government bodies for the needed legal and policy changes. For example, in Russia, PWID access to sterile injection equipment could be increased by inclusion of syringes in the federal list of mandatory medical products sold by pharmacies [18]. Arguments from public health and safety are likely to be the most persuasive and evidence should be marshaled from places that have successfully and effectively expanded pharmacy services such as needle/syringe sales.

Community-level stigma, discrimination, and resistance to harm reduction interventions also inhibit PWID’s access to all kinds of health services. Efforts are needed to convince community members that those suffering from drug addiction, a medical and psychosocial problem, would benefit from services delivered in a respectful and non-discriminatory manner and that such services contribute importantly to disease prevention without encouraging drug use.

In areas for potential expansion of pharmacy services in which there has been little success to date, such as vaccination, MMT/OST, and naloxone provision, stakeholders should assess the local situation and determine the importance of pharmacies providing these services before undertaking the difficult work needed to achieve the changes in the legal and policy framework that would be required. Laws limiting the liability of pharmacists who prescribe naloxone to treat opioid overdose were adopted in Massachusetts and Rhode Island since the completion of our study. There are also efforts underway to make naloxone available over the counter in the United States, but this would require approval of the Food and Drug Administration [http://drugtopics.modernmedicine.com/news/otc-naloxone-its-possible].

Advocates should consider multiple stakeholder viewpoints when designing policy reform efforts, including possible incentive mechanisms to engage pharmacies and other healthcare providers in services for drug users. No legal or policy changes appear to be necessary to allow expanded provision of counseling, informational materials, and referrals but structural changes such as provision of insurance coverage to compensate pharmacists for counseling or brief interventions may be needed to assure acceptability and sustainability of interventions [24,25].

Attention is also needed at the meso- and micro- levels. Laws and policies are only as good as their implementation. Serious gaps in the implementation of needle/syringe programs are well-documented and especially problematic in settings with weak rule of law [26-29]. Police department and pharmacy chain policies, and the knowledge, attitudes, and practices of individual PWID, individual pharmacies/pharmacists, and police officers should support rather than undermine extant laws and policies. Pharmacists’ attitudes and practices have been shown to influence provision of needles/syringes, opioid substitution treatment, counseling, and dissemination of HIV prevention and other health promotion materials to PWID in Scotland [30], Australia [31,32], and Estonia [9].

Training for pharmacists and police officers could be conducted to promote more supportive attitudes towards proposed programs [28,30,33]. Systematic monitoring of police abuse of drug users around pharmacies and other service points may also help address structural barriers to service access [34]. Programs are also needed to increase the self-efficacy of PWID to improve their relationships with pharmacists and thereby facilitate access to expanded services.

Overall, a considerable amount of advocacy will be needed if pharmacies are to reach their potential as venues for delivering services to drug users. This advocacy should employ a public health perspective, demonstrate cost-effectiveness of harm reduction interventions, and promote a human rights approach to the health problems of marginalized populations.

## Competing interests

The authors declare that they have no competing interests.

## Authors’ contributions

TH conceived the idea for the paper; SP, JG, PC, NZ, AL, AHK, EVF, RH, WS, RP, LB, CL, and DDJ contributed data and writing; all authors read and approved the final manuscript.

## Pre-publication history

The pre-publication history for this paper can be accessed here:

http://www.biomedcentral.com/1472-6963/14/261/prepub
